# Effect of Functionalization of Reduced Graphene Oxide Coatings with Nitrogen and Sulfur Groups on Their Anti-Corrosion Properties

**DOI:** 10.3390/ma14061410

**Published:** 2021-03-14

**Authors:** Karolina Ollik, Jakub Karczewski, Marek Lieder

**Affiliations:** 1 Department of Process Engineering and Chemical Technology, Faculty of Chemistry, Gdansk University of Technology, 11/12 Gabriela Narutowicza Street, 80-233 Gdansk, Poland; lieder@pg.edu.pl; 2 Department of Solid State Physics, Faculty of Applied Physics and Mathematics, Gdansk University of Technology, 11/12 Gabriela Narutowicza Street, 80-233 Gdansk, Poland; jakub.karczewski@pg.edu.pl

**Keywords:** heteroatoms, coatings, hydrophobicity, corrosion resistance

## Abstract

Electrophoretic production of anticorrosion carbonaceous coatings on copper could be successfully performed by anodic oxidation of negatively charged graphene platelets suspended in an aqueous solution. The various platelets were synthesized by Hummer’s method followed by a hydrothermal reduction in the presence of NH_4_SCN which was expected to substitute some parts of graphene structure with nitrogen and sulfur groups. X-ray photoelectron spectroscopy analysis confirmed that the graphene precursors, as well as the coatings, contained typical nitrogen groups, such as pyridinic and pyrrolic, and sulfur groups, such as thiol, thiophene, or C-SO_2_. However, due to oxidation during deposition, the qualitative and quantitative composition of the graphene coatings changed relative to the composition of the precursors. In particular, the concentration of nitrogen and sulfur dropped and some thiophene groups were oxidized to C-SO_2_. Studies showed the functionalized coatings had a uniform, defect-free, hydrophobic, more adhesive surface than nonmodified films. The corrosion measurements demonstrated that these coatings had better protective properties than the ones without these heteroatoms. This behavior can be assigned to the catalytic activity of nitrogen towards oxidation of C-SO_2_ groups to C-SO_3_H with oxygen.

## 1. Introduction

Graphene and its various oxidized or chemically functionalized forms have been recently studied as monolayers or multicomponent coatings for corrosion protection of metals. Graphene possesses properties that are beneficial for metal protection, such as chemical resistance, mechanical endurance, good adhesion to metals, or impermeability to gases and water. On the other hand, the conducting graphene layers might accelerate the corrosion in some instances [1]. Moreover, under ambient conditions, metal crystallographic orientation (especially for copper) is dominant to determine the degree of oxidation of substrate beneath graphene [2]. Deposition of the impeccable graphene coatings on a metal substrate is a rather challenging task. It can be carried out either from gaseous or aqueous phases, of which the first one, chemical vapor deposition, is essentially dedicated to the formation of (unoxidized) graphene layers [3,4,5]. Hence, the aqueous deposition seems to be more versatile, because the various chemically transformed entities of graphene, e.g., oxidized or doped, can be used as precursors, provided they are dispersible in water. The method of choice for this approach is electrophoretic deposition (EPD). It is a peculiarity of EPD that the composition of the coating differs from that of graphene precursor in a solution. One of the graphene precursors can be reduced graphene oxide (rGO). It is used, amongst other things, in photocatalytic hydrogen production and supercapacitors [6], electrochemical sensors [7], enhancement of four-electron transfer-assisted oxygen reduction, and methanol oxidation reaction [8]. Compared to rGO, graphene oxide is a more frequently used precursor in the fabrication of anticorrosion films. It is electrochemically active, and therefore, undergoes reduction (welcomed process) or oxidation (unwelcomed) at the electrode surface. Additionally, if the deposition takes place on an anode, some cations, due to limited metal dissolution, might be trapped in the graphene layer. Graphene oxide contains a relatively large amount of various oxygen groups, e.g., hydroxyl or carboxylic, which facilitate the dispersion in water. Some of these groups separate from the graphene skeleton as CO_2_ during electrodeposition [9]. This is an expected outcome from a corrosion protection perspective because the coating becomes less hydrophilic. On the other hand, the reduced content of oxygen in the coating does not lead to the restoration of an intact graphene structure (some carbon atoms in the evolution CO_2_ come from graphene structure). Nevertheless, the coatings probably contain a number of intact graphene domains, which might be current conductive. One can visualize such coatings as the one composed of three domains: pure graphene (current-conducting), oxidized graphene (hydrophilic, but still impermeable to oxygen or water), and defects or holes that do not protect the metal surface. Once graphene oxide is employed as the EPD precursor, we end up with coatings that do not meet the criteria of good protective properties [10,11,12]. There are at least two techniques for solving this problem. First, we consider multilayer (consecutive) deposition, a well-known protocol adopted to tackle the porosity of nickel coatings, as not scientifically challenging and, second, a substitution of a small percentage of carbon atoms in the graphene precursors with nitrogen. There are numerous published reports confirming the presence of pyridinic, pyrrolic, and other nitrogen-based groups in N-substituted graphene. Kumar et al. reported corrosion resistance results from hydrophobic and intact surfaces compared to rGO and GO coatings [13]. In turn, in another case, the good protective properties of nitrogen-doped coatings were caused by low conductivity and continuous surfaces [14]. However, these structures were supposed to catalyze oxygen reduction [15], which they seemingly did, a role we do not expect nitrogen to play as far as the protective layers are concerned. We decided to follow this route because nitrogen groups might add a promising feature to the graphene coatings—i.e., disruption of electron transfer across the graphene domains. Our expectations were partly confirmed. N-substituted graphene oxides produced coatings that performed better than unsubstituted ones. However, in the conclusion, we write that protection would have been better if nitrogen groups had not catalyzed oxygen reduction [16]. Indeed, we found that the presence of nitrogen groups in the graphene coatings is generally beneficial for corrosion protection, but still, we have tried to figure out how to block the noxious effect of nitrogen catalytic activity. We came up with the idea of sulfur codoping because there were a couple of reports on catalytic oxidation of gaseous SO_2_ to SO_3_ over N-doped graphene [17,18]. We hoped that it would be also possible to oxidize C-SO_2_ to C-SO_3_H with oxygen (from the air) penetrating the N-doped graphene coatings. As a matter of fact, this reaction also involves oxygen reduction, but the electrons are donated by sulfur not metal, thus it may diminish corrosion.

Nitrogen- and sulfur-functionalized reduced graphene oxide coatings as the anti-corrosion layer have not been reported yet. In the present work, graphene oxide, reduced graphene oxide, and nitrogen- and sulfur-functionalized reduced graphene oxide were synthesized by an improved Hummer’s method, one-step hydrothermal method, and one-step hydrothermal method in presence of ammonium thiocyanate, respectively. Subsequently, the obtained products were electrophoretically deposited on the copper substrate and subjected to numerous studies, revealing their properties.

## 2. Materials and Methods

### 2.1. Reagents

Copper sheets (purity: 99.99%, size 10 × 15 mm, thickness: 1 mm, not annealed), graphite powder (325 mesh, purity: 99.9995%, Alfa Aesar, Haverhill, MA, USA), potassium permanganate (KMnO_4_, purity: 99.0%, POCH, Gliwice, Poland), orthophosphoric acid (H_3_PO_4_, 85%, POCH), sulfuric acid (H_2_SO_4_, 95%, POCH), hydrogen peroxide (H_2_O_2_, 30%, CHEMPUR, Piekary Slaskie, Poland), ethanol (C_2_H_5_OH, 96%, POCH), hydrochloric acid (HCl, 35–38%, POCH), acetone ((CH_3_)_2_CO, POCH), ammonium thiocyanate (NH_4_SCN, purity: 99.0%, POCH), and sodium chloride (NaCl, purity: 99.5%, POCH) were used. 

### 2.2. Synthesis of Graphene Oxide

Graphene oxide was synthesized by the improved Hummer’s method [19]. Concentrated sulfuric and phosphoric acids in a ratio of 9:1 were added to a round bottom flask containing 6 g KMnO_4_ and 1 g graphite powder. Then, the solution was heated to ca. 70 °C and stirred overnight. After cooling to room temperature, the solution was mixed with 200 g ice and 1 mL H_2_O_2_, then filtrated under vacuum and finally washed with 250 mL water, 250 mL concentrated HCl, and two times with 250 mL ethanol. After each washing step, the solid was separated by centrifugation (6000 rpm for 15 min). At the end, the precipitate was washed with acetone, which was then evaporated, leaving around 1 g of a brown solid.

### 2.3. Synthesis of Reduced Graphene Oxide

The one-step hydrothermal method was used for the preparation of reduced graphene oxide [20]. In total, 200 mg GO was dispersed in 40 mL of distilled water by using an ultrasonic bath for 30 min. Then, the solution was placed in stainless steel autoclave and subjected to a hydrothermal reaction at 180 °C for 6 h. After cooling to room temperature, the solution was filtrated under vacuum and washed several times with distilled water. Finally, the solid was dried in the oven at 60 °C for 24 h.

### 2.4. Synthesis of Nitrogen- and Sulfur-Functionalized Reduced Graphene Oxide

Nitrogen- and sulfur-functionalized reduced graphene oxides (N,S-rGO) were prepared by the one-step hydrothermal method in the presence of ammonium thiocyanate [21]. GO (200 mg) and NH_4_SCN (300, 450, and 600 mg, samples were labeled as N,S-rGO300, N,S-rGO450, N,S-rGO600, respectively) were dispersed in 40 mL of distilled water by using an ultrasonic bath for 30 min. Then, the mixture was put into a stainless steel autoclave heated to 180 °C for 6 h. Afterwards, the solid was filtered under vacuum, washed several times with distilled water, and dried at 60 °C for 24 h.

### 2.5. Electrophoretic Deposition on the Copper Substrate

Prior to EPD, the copper substrate was properly prepared by the removal of contaminations and copper oxides using hydrochloric acid, distilled water, and acetone. Then, GO, rGO, N,S-rGO precursors of coatings were dispersed in distilled water by using the ultrasonic bath for 0.5, 1.5, and 2 h, respectively. The experimental setup for EPD was made of two parallel arranged copper electrodes separated from each other by around 1 cm. The electrodes were connected to a power supply. This process was carried out using the following parameters: applied voltage 15 V, time of deposition 15 s, and a suspension concentration of 0.5 mg mL^−1^. After the electrolysis, the anodes were air-dried.

### 2.6. Characterization

All functionalized precursors of coatings were prepared from newly synthesized graphene oxide. Results for GO and rGO are meant to be reference data. Fourier Transform Infrared Spectroscopy (FTIR) of precursors of the coatings was conducted on the spectroscope PerkinElmer Frontier over a scanning range 550–4000 cm^−1^. X-ray diffraction (XRD) analysis was performed with Rigaku MiniFlex 600 in the range of angle 2°–90° (Cu Kα radiation, *λ* = 1.54 Å). Characteristic crystallographic parameters such as an interlayer distance (*d*_002_), an in-plane crystallite size (*L_a_*), and an average crystallite width (*L_c_*) were calculated using the following equations:

(1) $$d_{002}=\;\lambda /2\cdot sin\theta_{1}$$

(2) $$L_{a}=\;1.84\cdot \lambda /FWHM\cdot cos\theta_{2}$$

(3) $$L_{c}=\;0.89\cdot \lambda /FWHM\cdot cos\theta_{1}$$

*λ*—radiation wavelength (Å);
*θ*_1_—(0 0 2) diffraction peak position (°);
*θ*_2_—(1 0 0) diffraction peak position (°);
*FWHM*—width at half height of the corresponding diffraction peak (rad).

Raman spectra were recorded on Horiba-Jobin Yvon microprobe apparatus excited by a 532 nm laser. A ratio of I_D_/I_G_ was calculated as the quotient area under the D peak and under the G peak. As a result of the deconvolution of bands, the area under peaks was obtained. X-ray photoelectron spectroscopy (XPS) analysis was prepared by the Omicron NanoTechnology system at a pressure below 1.1 × 10^−8^ mBa and at room temperature. The Argus hemispherical spectrometer with a 128-channel collector was used for the energy measurement of photoelectrons. The photoelectrons were excited by a Mg-Kα X-ray source operated at 15 keV and 300 W. Analysis of obtained results was performed using CASA XPS software package with Shirley background subtraction and least-square Gaussian–Lorentzian—GL(30) curve fitting algorithm. Calibration of measured spectra to the binding energy of 285 eV for the C1s line was conducted. Zeta potential measurements of the graphene sheets in aqueous suspensions were carried out by using Malvern Zeta-Sizer. A contact angle of the coatings was determined by the goniometer Cam 200 KSV. The used liquid was distilled water (dosing volume of water of 1 µL). The surface topography of layers was defined by the Scanning Electron Microscope (SEM) and Atomic Force Microscopy (AFM). SEM images before the electrochemical tests were collected on the microscope Quanta FEG 400-FEI Company (SE detector, 15 kV beam accelerating voltage), whereas SEM images after electrochemical tests and thickness of the coatings were obtained by SEM Microscope FEI Quanta FEG 250 with a SE-ETD detector (secondary electron—Everhart-Thornley detector), using 10 kV beam accelerating voltage. Film thickness is the average value of all thickness measurements of the layers. AFM images were obtained using Bruker Catalyst by noncontact mode using Au coated silicon tips (scan rate—0.273 Hz). The cross-cut test was used to determine adhesion between the graphene coating and the metal substrate. With a knife, cuts separated by about 1 mm were made on the surface of coatings. After an adhesive tape was broken, on the basis of the number of detached graphene flakes, the adhesion of coatings was determined.

### 2.7. Electrochemical Studies

A sodium chloride solution (3.5% NaCl) was used as the corrosive medium. Chloride ions are very aggressive for the copper substrate; therefore, it is important to assess the durability of the coatings in this environment. For evaluation of the protective properties of the graphene coatings, a potentiodynamic polarization was applied. Measurements were carried out by using a potentiostat, ATLAS-SOLLICH 0531 (Poland), connected to a cell containing the coated copper substrate as a working electrode, an Ag/AgCl (KCl saturated) reference electrode, and a platinum wire as a counter electrode. Electrochemical tests were performed twice for each sample, in a potential range of −0.1 to 0.1 V (vs. open circuit potential) with a scan rate of 0.5 mV s^−1^. To stabilize potential, every sample was immersed in the solution for 30 min before measurement.

Corrosion potential (*E_corr_*) and corrosion current density (*I_corr_*) were determined from the intersection of the anode and cathode curves. Corrosion rate (*CR*) was calculated from the following equation (ASTM G59):

(4) $$CR=K\cdot I_{corr}\cdot \frac{EW}{d}$$

*K*—corrosion rate constant (mm year^−1^);
*I_corr_*—corrosion current density (µA cm^−2^);
*EW*—equivalent weight, for Cu 31.7 g;
*d*—density, *d*_Cu_ = 8.97 g cm^−3^.

## 3. Results

**Scheme 1:**
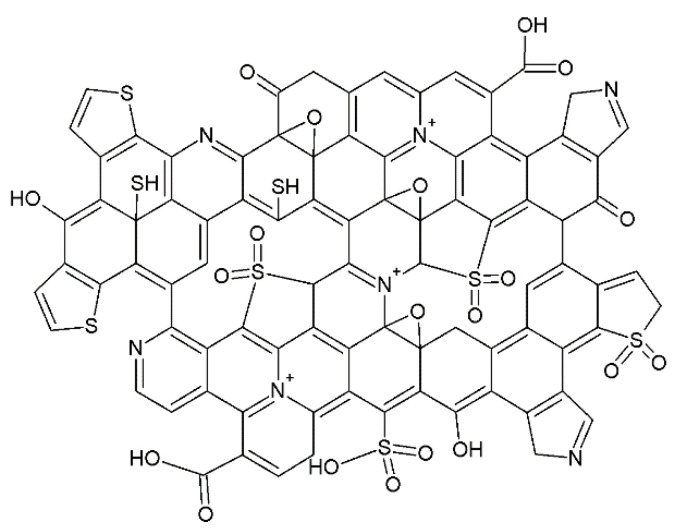
Structure of nitrogen and sulfur functionalized reduced graphene oxide.

Functionalization of graphene oxides samples was carried out by prolonged reacting them with ammonium thiocyanate at 180 °C. Due to thermal instability, this compound decomposed to NH_3_, CS_2_, H_2_S, and HSCN [22], leading to a substitution of carbon and oxygen atoms in the basic structure of GOs with nitrogen and sulfur moieties. Ammonia contributed to the formation of pyridinic and pyrrolic sites [23], whereas H_2_S as a sulfurizing agent created thiophene and thiol sites [24,25]. Moreover, oxygen released during the hydrothermal process caused oxidation of sulfur in these sites to C-SO_2_, or C-SO_3_H [26,27] (Scheme 1).

**Figure 1:**
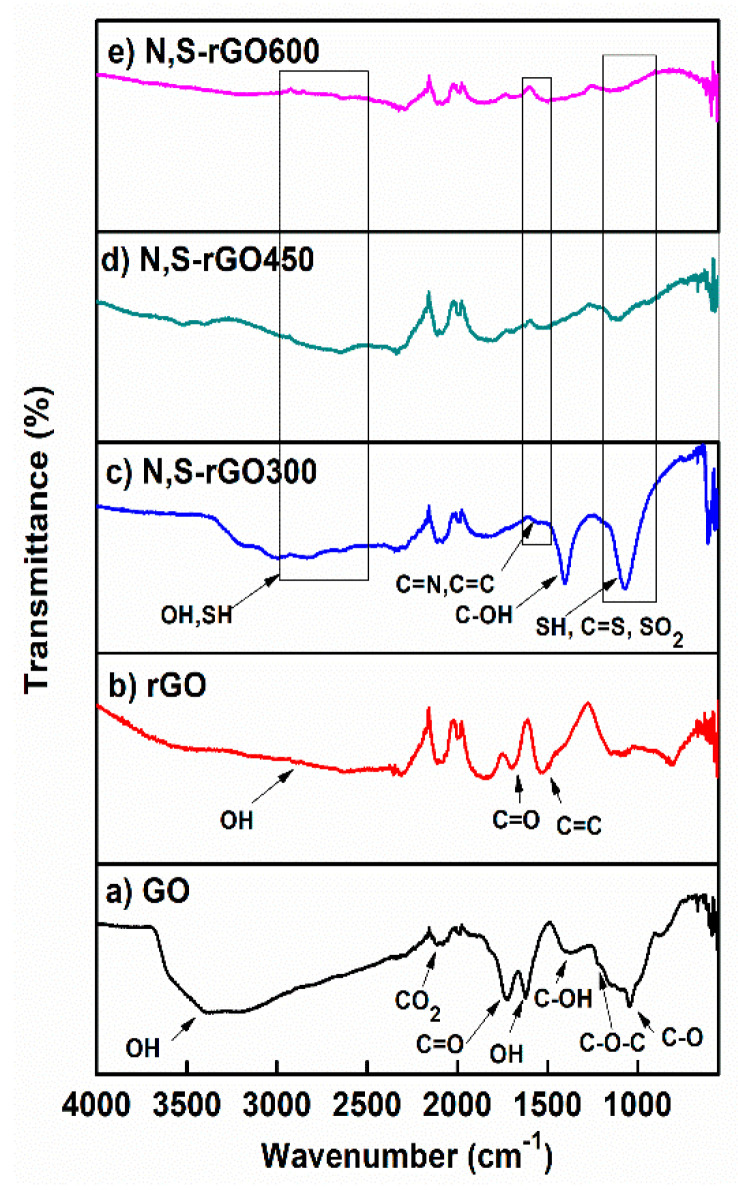
Fourier Transform Infrared Spectroscopy (FTIR) spectra for precursors of coatings. (**a**) GO, (**b**) rGO, (**c**) N,S-rGO300 (**d**) N,S-rGO450 (**e**) N,S-rGO600.

There is only one visible band, around 1500 cm^−1^, that could be clearly attributed to C=N bonds. However, this very weak signal is apparently convoluted with the one attributed to C=C bonds. This supposition was confirmed by the spectra of GO and reduced graphene oxide (rGO). The stretching energy of 1500 cm^−1^ is completely missing for GO (Figure 1a) because there are no sp^2^ domains in this structure. As the sp^2^ domains are restored by thermal reduction (rGO), the strong band at 1500 cm^−1^ shows up (Figure 1b). When the reduction of GO is conducted in the presence of ammonia, then the 1500 cm^−1^ band is also detectable in the spectra [9]. This time its intensity is weaker, probably due to the lower content of the C=C domains, which could not be made up by the presence of C=N bonds. The same bands are shown in Figure 1c–e become very minute due to further decrease in the C=C content, which we relate to the presence of sulfur in addition to nitrogen atoms in the structure of the reduced graphene. The presence of some sulfur groups reveals the band at 1070 cm^−1^, in particular C=S stretching vibrations, symmetrical stretching SO_2_ bands, and SH deforming vibrations. The intensity of this band is the highest for N,S-rGO300, and the lowest for N,S-GO600. The thiol groups should also give rise to a peak at 2600 cm^−1^, however, it was overlapped by a wide OH band.

**Figure 2:**
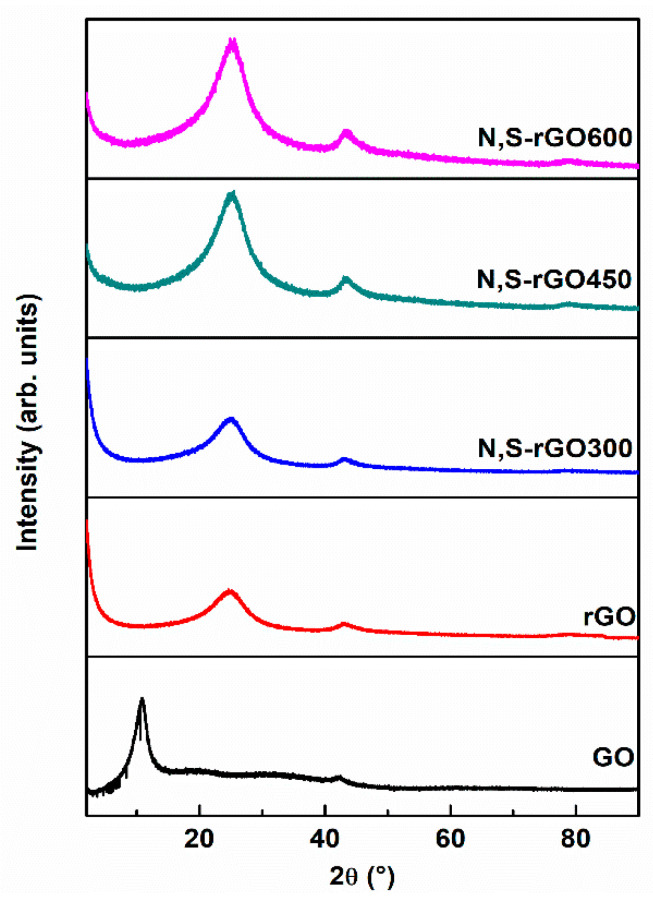
XRD patterns for precursors of coatings.

During hydrothermal processes of sample preparation, thermal energy is sufficient to break the majority of C-O bonds and replace some of them with C-N or C-S bonds, allowing the distance to be shortened between graphene sheets and to preserve their parallel orientation. Well-resolved and sharp XRD reflections, shown in Figure 2, could be related to these reasonably ordered interlayer graphene packings. The lateral extent of the graphene layers (L_a_) was estimated from the width of (1 0 0) reflections at ca. 43°, while the stacking number of graphene sheets (L_c_) was estimated from the width of (0 0 2) peaks at ca. 26° (Table 1). One might have expected that the bulky sulfur groups would have helped to preserve the large distance between the graphene sheets; however, as the data show, it did not happen because the sulfur groups are mainly located near a periphery of the graphene structures (Scheme 1). There are a relatively large number of such sites due to the cleavage in the basal plane of GOs during the hydrothermal reductions as a result of an evolution of CO and CO_2_ [28] (lower lateral dimension) resulting in an increase in the edge-to-surface ratio.

**Figure 3:**
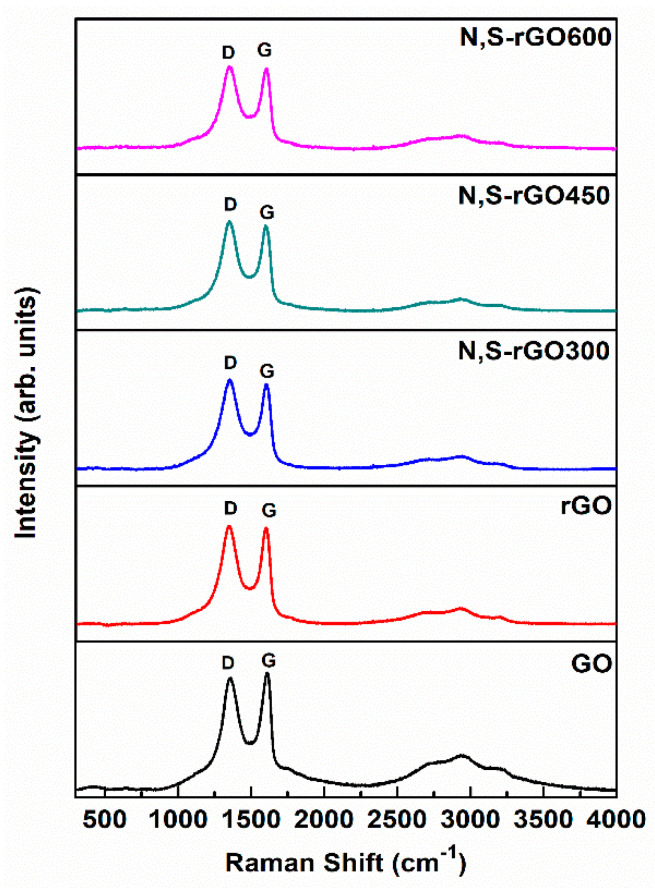
Raman spectra for precursors of coatings.

The observations were further verified by Raman spectroscopy (Figure 3). The N,S-rGO300 samples have the highest I_D_/I_G_ ratio, which confirms the largest concentration of internal and peripheral edges in the plane of these graphene platelets (Table 2). Moreover, the introduction of heteroatoms to the structure of graphene oxide also contributed to the increasing ratio compared to the nonmodified precursors of coatings. 

The elemental compositions of the studied graphene precursors and coatings were studied by the XPS technique (Figures S1 and S2). The high-resolution C1s spectra of the samples functionalized with nitrogen and sulfur were assigned to C=C (~284.59 eV), C-N/C-S/C-O (hydroxyl and epoxy bonds) (~285.44 eV), C=O (~287.76 eV), and O-C=O (~289.58 eV) [29]. The high-resolution N1s spectra show three peaks at ~397.73, ~399.48, ~401.00 eV, which correspond to pyridinic-N, pyrrolic-N, and graphitic-N, respectively [30,31]. The spectra of S2p are composed of three components, which are attributed to thiolic-S [32] and thiophenic-S [33] and oxidized forms of C-SO_x_ (x = 2,3) [29]. 

The percentage chemical compositions of the precursors and coatings are presented in Table 2. These results show that during the hydrothermal reduction, the deoxygenation of the samples took place, which resulted in a partial restoration of the sp^2^ hybridization of the carbon atoms (see Figure 1—intensity of functional oxygen groups peaks was significantly decreased). These results, relevant to the GO and rGO samples, are in accordance with the one we have recently published [16]. Moreover, the analysis of the results also shows that the deoxygenation of the graphene platelets deepens during the formation (electrodeposition) of the coatings. This is probably due to the oxidative Kolbe’s reaction, simultaneously causing an increase in the concentration of nitrogen compared to precursors of coatings. On the other hand, as expected, the concentration of heteroatoms in the structure of functionalized graphene platelets increases with concentration of ammonium thiocyanate used for the synthesis (an increase in the I_D_/I_G_ ratio for the functionalized samples in comparison to nonmodified samples). During electroformation of the coatings, many thiophene and thiols were oxidized to carbon bound –SO_2_ groups, although some sulfur was lost due to SO_2_ gas release.

Electrophoretic deposition requires stable dispersions. Due to the large interlayer distance for graphene oxide maintained by weak interactions, the stable suspension of graphene oxide was easily produced by 0.5 h treatment in an ultrasonic bath. Reduced graphene oxide samples were characterized by significantly lower interlayer spacings and thus, stronger interactions between layers. This caused the obtention of uniform suspension more difficult, simultaneously contributing to prolonged ultrasonic treatment (around 2 h). The obtained suspensions were stable due to the Zeta potential (ζ) being lower than −30 mV [34]. Measured potentials were −47.6, −41.4, −59.5, −55.7, and −50.5 mV for GO, rGO, N,S-rGO300, N,S-rGO450, and N,S-rGO600, respectively. 

As expected, the Zeta potential for rGO is higher (less negative) than that for GO. However, in the case of functionalized graphene oxide samples, the Zeta potential is much lower than that for GO and rGO due to the presence of the C-SO_2_ groups. Moreover, an increase in the potential from N,S-rGO300 to N,S-rGO600 samples results from the increasing content of graphitic nitrogen (0.9%, 1.2%, and 3.0% for N,S-rGO300, N,S-rGO450, and N,S-rGO600), whose positive charge compensates the negative charge of C-SO_2_ groups.

**Figure 4:**
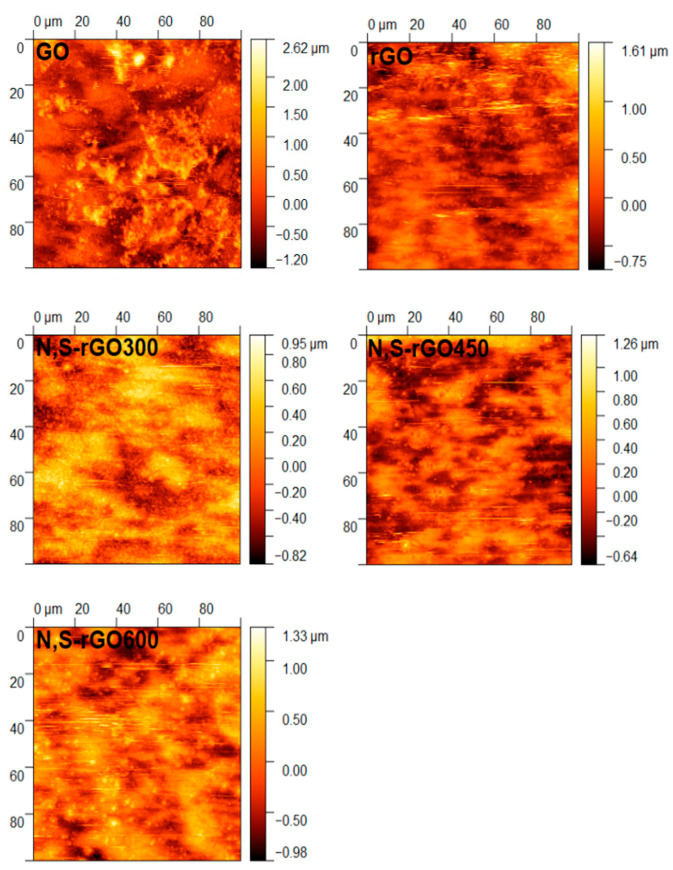
Atomic Force Microscopy (AFM) images for graphene coatings.

The roughness of the coatings was estimated by the AFM (Figure 4 and Table 3). The results show that the coatings obtained from the GO precursor were the most rough, probably due to intensive CO_2_ release during electrolysis. The other precursors were not subjected to the destructive CO_2_ evolution because they contained a substantially lower concentration of oxygen in their structures. The same observation is also true for the functionalized coatings. 

**Figure 5:**
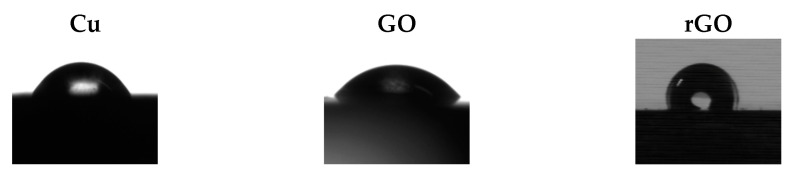

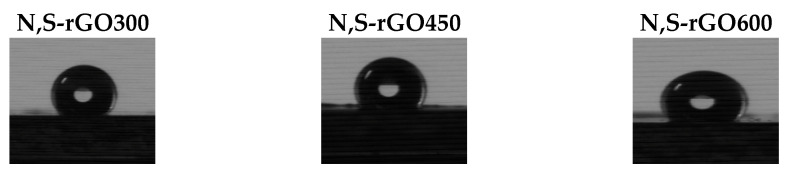
The contact angle images.

The low wettability of the protective coatings is a crucial property to fend off water. The measured contact angles show (Table 3 and Figure 5) that all functionalized coatings are highly hydrophobic and should effectively repel water from their surface.

**Figure 6:**
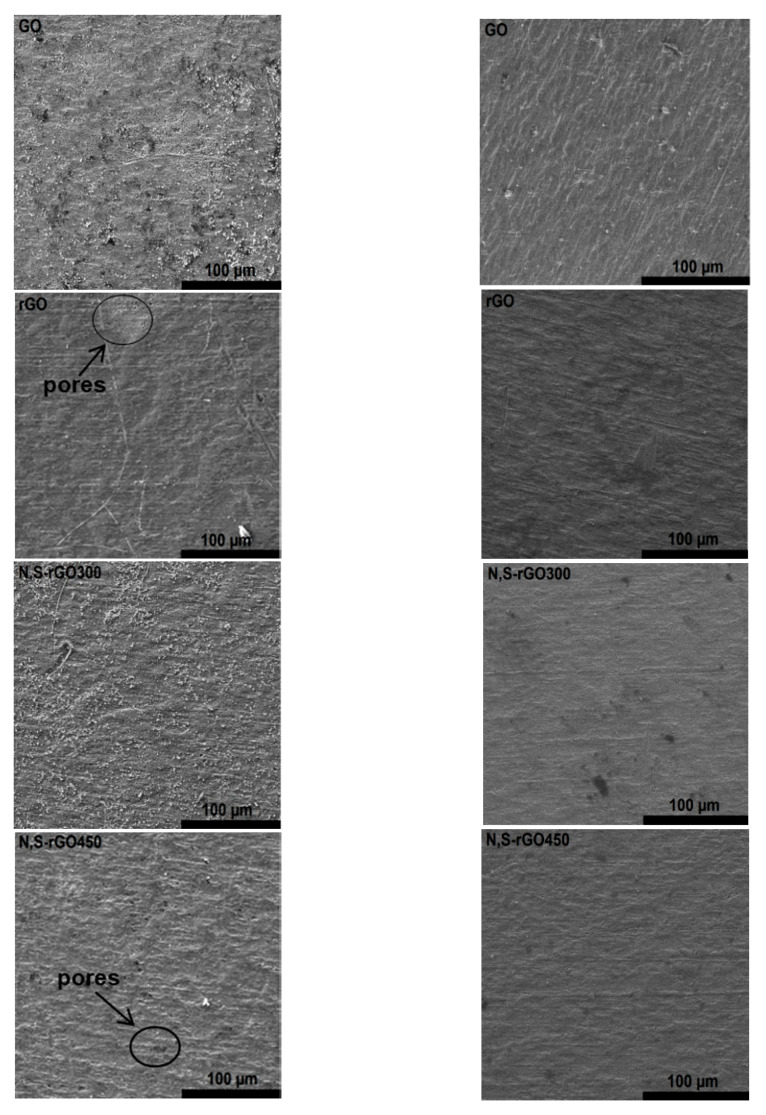

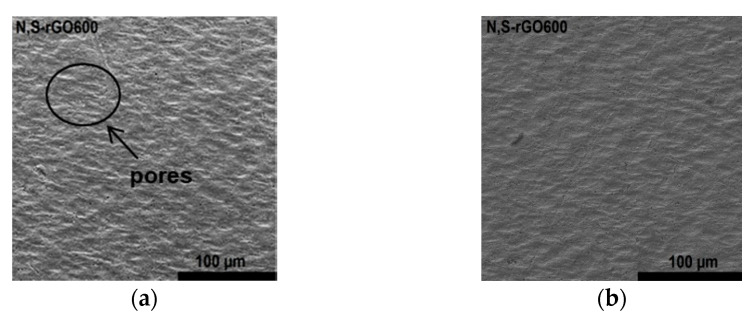
Scanning Electron Microscope (SEM) images for graphene coatings: (**a**) before corrosion test; (**b**) after corrosion test.

**Figure 7:**
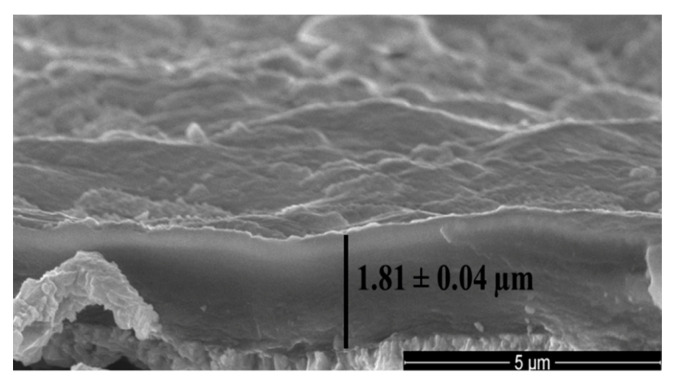
The thickness of graphene coatings.

**Figure 8:**
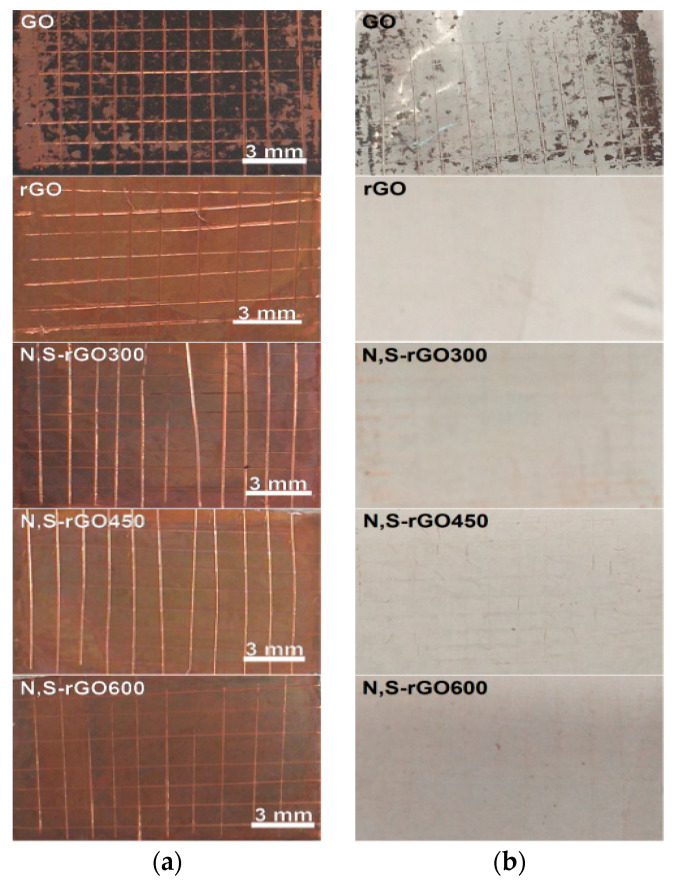
Photographs of graphene coatings: (**a**) after cross-cut test; (**b**) view of tape after breaking from coatings.

SEM images of all coatings, before and after electrochemical studies, are presented in Figure 6. In general, their surfaces were smooth and uniform with a few wrinkles or minute pores. The average thickness of all coatings was 1.81 ± 0.04 μm (Figure 7). The coatings were subjected to partial oxidation due to anodic polarization, which resulted in their further smoothing down. Figure 8 also shows the cross-cut adhesion test results for the coatings. The large number of detached flakes indicates that the GO coating was the least adhesive to the copper substrate, whereas the other coatings possessed reasonable surface grip to the metal. 

**Figure 9:**
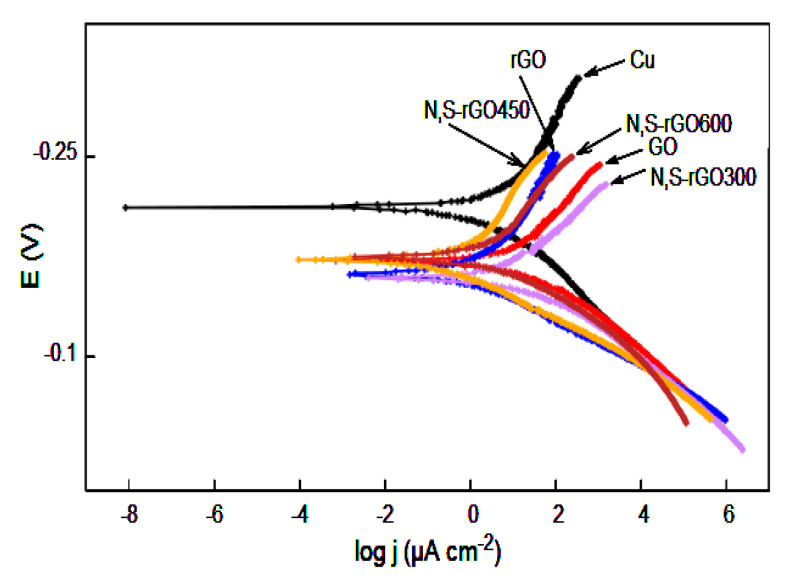
Polarization curves for copper and graphene coatings.

The corrosion resistance of all coating against 3.5% sodium chloride solution was tested by a potentiodynamic polarization method. The results are presented by the Tafel plots in Figure 9, and the Tafel parameters are shown in Table 4. 

As can be seen in Figure 9, all coated samples reduced electrochemical activity. The *E_corr_* shifted towards more positive values for all coated samples, indicating that coatings probably act as a barrier separating copper from the aggressive environment. However, the hydrophilic nature of the graphene oxide allows penetration of the GO coatings by water, causing oxidation of copper. Moreover, the high current density stems also from the partial or complete oxidation of carbon, which may finally result in the decomposition of the coatings.

C-C + 2H_2_O → OH-C-C-OH + 2H^+^ + 2e^−^

C + 2H_2_O → CO_2_ + 4H^+^ + 4e^−^

In the case of the rGO coatings, their hydrophobicity makes them an impenetrable water barrier. Therefore, the measured corrosion current stemmed only from the oxidation of carbon. Thanks to this, the surface of these coatings became smoother after the electrochemical tests.

**Figure 10:**
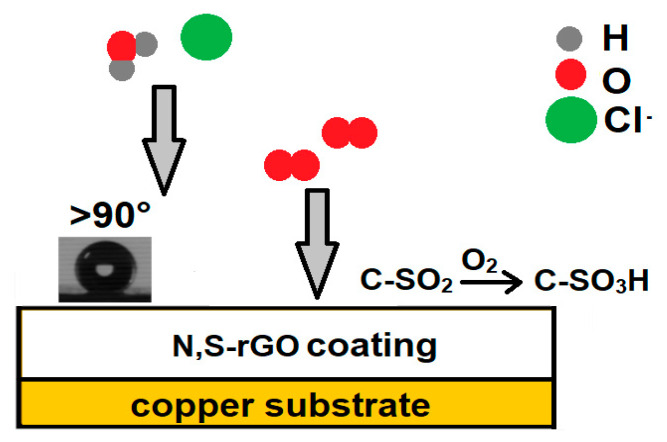
The protection mechanism of functionalized graphene coatings.

In our previous work, we showed that the nitrogen-functionalized graphene oxide coatings had protective properties, but these were comparable to the anticorrosion properties of the reduced graphene oxide coatings. Nitrogen groups exhibiting catalytic properties towards the oxygen reduction reaction were responsible for this behavior. The coatings produced in this study contain a substantial amount of C-SO_2_ groups formed as a result of the oxidation of thiophene groups during EPD. Nitrogen that is also present in the structure can effectively catalyze the oxidation of these groups to C-SO_3_H by oxygen from the air, so oxygen can no longer depolarize the copper metal. The corrosion protection mechanism of the graphene coatings is presented in Figure 10.

## 4. Conclusions

The introduction of nitrogen and sulfur groups to the graphene oxide structure contributed to the improved corrosion resistance of graphene coatings produced by EPD on the copper substrate. These coatings had numerous C-SO_2_ groups in their structures, which formed as a result of the oxidation of thiophene groups. Nitrogen can effectively catalyze the oxidation of these groups to C-SO_3_H with oxygen from the air. 

## Figures and Tables

**Table 1:** X-ray diffraction (XRD) parameters for precursors of coatings.

Precursor of Coating	2Ѳ (°) (002)	d (Å)	L_c_ (Å)	2Ѳ (°) (100)	L_a_ (Å)	I_D_/I_G_
GO	10.74	8.19	24.14	42.30	121.63	1.64
rGO	24.64	3.62	11.40	42.99	89.68	1.98
N,S-rGO300	24.62	3.62	11.59	43.06	86.93	2.11
N,S-rGO450	24.74	3.60	10.79	43.30	109.00	1.92
N,S-rGO600	24.87	3.58	11.13	43.28	89.68	1.94

**Table 2:** XPS estimate of the surface relative C, O, N and S content of the precursors of coatings and coatings.

	Precursor of Coating				Coating			
	C (%)	O (%)	N (%)	S (%)	C (%)	O (%)	N (%)	S (%)
GO	41.7	58.3	-	-	51.5	48.5	-	-
rGO	66.8	33.2	-	-	69.6	30.4	-	-
N,S-rGO300	66.7	29.8	1.6	1.9	60.5	33.4	5.2	0.9
N,S-rGO450	67.5	28.5	1.9	2.1	61.6	34.0	3.6	0.8
N,S-rGO600	66.6	26.8	3.7	2.9	61.9	34.3	3.5	0.3

**Table 3:** Contact angle values and surface roughness parameters for graphene coatings.

Coating	Contact Angle Value (°)	Surface Roughness Parameters	
		Sq (nm)	Sa (nm)
Cu	58.80 ± 0.70	-	-
GO	59.50 ± 2.25	478.1	366.0
rGO	109.23 ± 1.08	278.7	219.3
N,S-rGO300	141.07 ± 1.87	267.7	222.2
N,S-rGO450	119.06 ± 0.98	261.5	215.8
N,S-rGO600	133.97 ± 1.28	311.2	254.3

**Table 4:** Corrosion parameters for bare copper and graphene coatings.

Coating	I (µA cm^−2^)	E (V)	Corrosion Rate (mm year^−1^)
Cu	4.42	−0.220	0.051
GO	11.12	−0.188	0.128
rGO	1.90	−0.152	0.022
N,S-rGO300	7.28	−0.182	0.084
N,S-rGO450	0.16	−0.182	0.002
N,S-rGO600	0.90	−0.186	0.010

## Data Availability

Data are contained within the article and supplementary material.

## Supplementary Materials

The following are available online at https://www.mdpi.com/1996-1944/14/6/1410/s1: Figure S1: XPS spectra for precursors of coatings. Figure S2: XPS spectra for graphene coatings.
