# Supplementary material for: Effect of Functionalization of Reduced Graphene Oxide Coatings with Nitrogen and Sulfur Groups on Their Anti-Corrosion Properties
Source: Materials (Basel). 2021 Mar 14;14(6):1410. doi: 10.3390/ma14061410 (PMC8001060; doi:10.3390/ma14061410)
Supplement: Supplementary file 1 [file materials-14-01410-s001.pdf]

Effect of functionalization of reduced graphene oxide coatings with nitrogen and sulfur groups on their anti-corrosion properties

Karolina Ollik [1]*, Jakub Karczewski [2], Marek Lieder [1]

[1] Department of Process Engineering and Chemical Technology, Faculty of Chemistry, Gdansk University of Technology, 11/12 Gabriela Narutowicza Street, 80-233 Gdansk,
[2] Department of Solid State Physics, Faculty of Applied Physics and Mathematics, Gdansk University of Technology, 11/12 Gabriela Narutowicza Street, 80-233 Gdansk,
* Correspondence: karolina.ollik@pg.edu.pl; 58 347 12 75

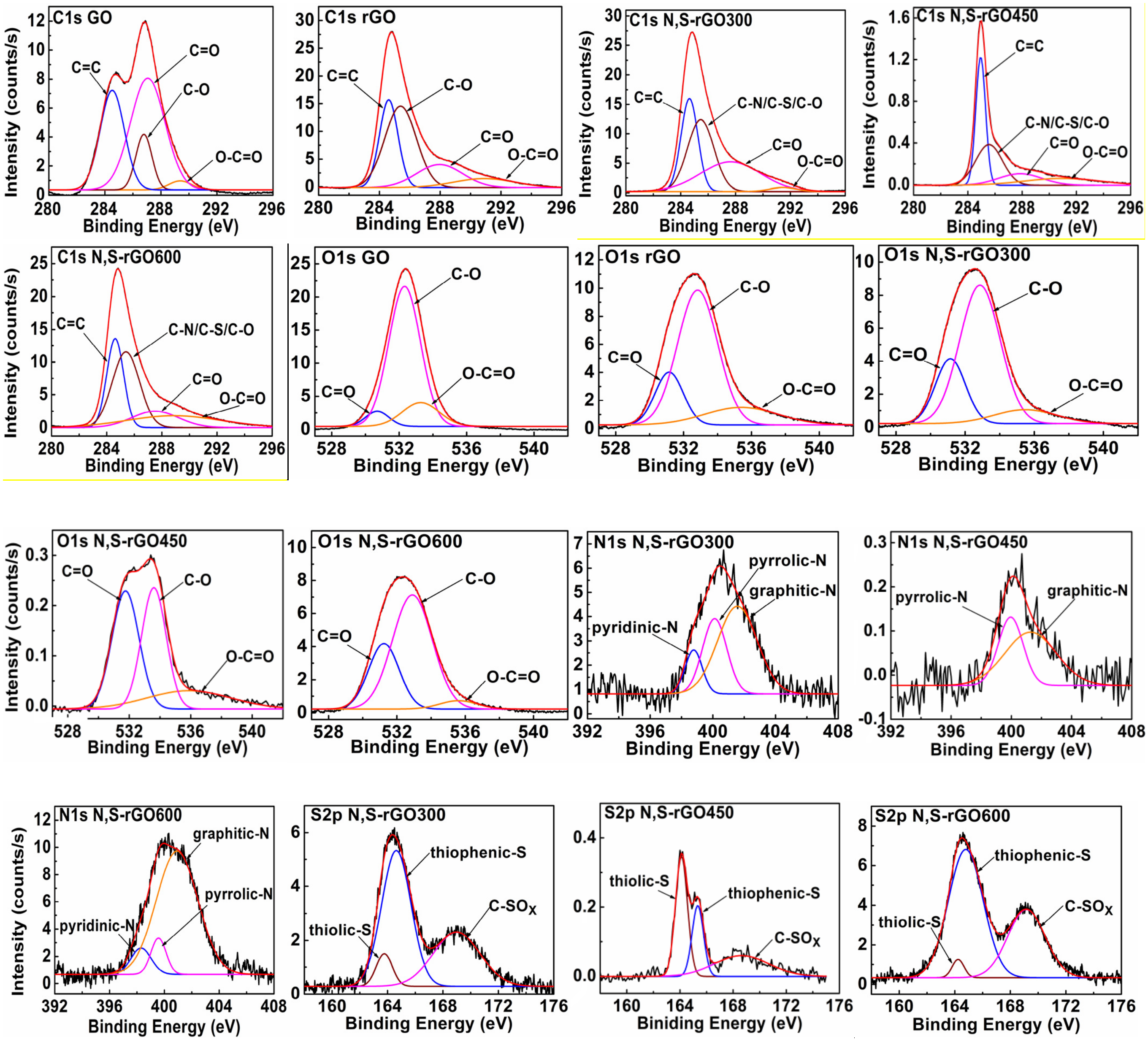

Figure S1. XPS spectra for precursors of coatings.

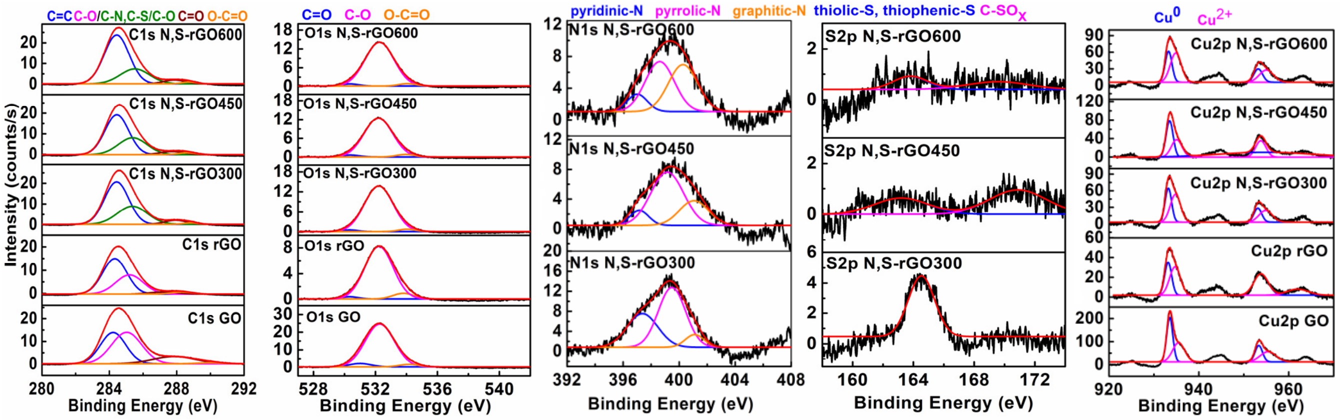

Figure S2. XPS spectra for graphene coatings.
